# Understanding factors that affect wellbeing in trans people “later” in transition: a qualitative study

**DOI:** 10.1007/s11136-022-03134-x

**Published:** 2022-04-12

**Authors:** Z. Aldridge, N. Thorne, E. Marshall, C. English, A. K. T. Yip, E. Nixon, G. L. Witcomb, W. P. Bouman, J. Arcelus

**Affiliations:** 1 Institute of Mental Health, Faculty of Medicine and Health Sciences, University of Nottingham, Nottingham, UK; 2 Nottingham Centre for Transgender Health, Nottingham, UK; 3 School of Sport, Exercise and Health Sciences, Loughborough University, Loughborough, Leicestershire LE11 3TU UK; 4 Gendered Intelligence, VAI, 200a Pentonville Road, London, N1 9JP UK

**Keywords:** Wellbeing, Life satisfaction, Transgender, Gender affirming medical treatment, Qualitative

## Abstract

**Purpose:**

Although cross-sectional studies have demonstrated that trans people present with lower quality of life and wellbeing than the general population, few studies have explored the factors associated with this, particularly in those who have medically transitioned some time ago. This paper aims to fill the gap in the literature on what factors are associated with wellbeing in trans people who initiated medical transition some time ago.

**Methods:**

This study used semi-structured one-to-one interviews with 23 participants to investigate the factors that impact upon the wellbeing of trans people who had initiated Gender Affirming Medical Treatment five or more years ago. The content of the interviews were analysed with an inductive, grounded theory approach to identify common themes within them.

**Results:**

The four themes identified include some consistencies with cisgender populations (while being viewed through the lens of trans experience), as well as those more specific to the trans experience. Together these themes were*: Interactions with healthcare services; Seeking societal acceptance; Quality of social support; The ‘double-edged sword’ of media and social media*. Each of the themes identifies a factor that participants highlighted as impacting, either positively or negatively, on their wellbeing.

**Conclusions:**

The results highlight the importance of social support, protective legislations, awareness of trans issues in the general public, and the need of improving the knowledge held by non-specialist healthcare providers.

## Introduction

Transgender, or trans, covers several gender identities, including trans women (those who were assigned male at birth but identify as women), trans men (those who were assigned female at birth but identify as men) and gender diverse people (those who identify and/or present outside the gender binary) [1–4]. Some trans people wish to have Gender Affirming Medical Treatment (GAMT; which may include Gender Affirming Hormone Treatment [GAHT] and/or Gender Affirming Surgery [GAS]), while others do not.

Research in the field of trans wellbeing identifies that trans people tend to have poorer quality of life (QoL) and psychological wellbeing than the general population [5–9], with better QoL linked to GAMT [1, 4]. Cross-sectional quantitative studies, with mainly clinical populations, have found that some of the factors associated with improved wellbeing in trans people included social and family support, being in employment, being highly educated, having a high household income, having a partner, and being younger [5–9]. On the other hand, anxiety, poor sleep quality, experiencing pain, reduced self-esteem, and having high interpersonal issues were factors associated with poorer wellbeing and QoL [5–9]. Some of these factors are similar to those found in studies examining wellbeing in cis people [10–14]. However, these factors have different nuances when examined through the lens of trans experience and when inextricably linked with being trans (e.g., transphobic discrimination influencing employment, loss of support upon coming out). In addition to those factors that are seen in both cis and trans populations there are issues such as legislation which are specifically experienced by trans people. For example, a report by the European Commission in 2020 stated that the UK imposed intrusive medical requirements for trans people to have legal gender recognition [15].While the findings of these studies have provided valuable insight, the information is predominantly based on trans people attending trans health services (THS), most of whom are at the early stages of clinical transition [16]. However, there is a lack of information regarding the wellbeing of trans people who have initiated GAMT some time ago—many of whom are no longer in touch with, or have very limited contact with, THS. This information is vital to provide a deeper understanding of the issues that impact wellbeing in this under-researched population and, if needed, improve the long-term outcome of any gender affirming intervention.

Therefore, this study aims to fill this gap by exploring the factors, aside from the effect of medical transition, that affect the wellbeing of trans people five or more years post initiation of GAMT. This timeline was chosen as many trans people are not in contact with THS by then. Since medical transition has different meanings for different people, it can be considered as a continuum, and for some, it can be seen as having no end date. For the context of this paper, people who have initiated GAMT over five years ago will be regarded as being at a stage ‘later in transition’.

## Methods

### Design

A qualitative approach was adopted to gain information about the factors influencing the wellbeing of trans people. Semi-structured interviews were conducted either face-to-face or via video call, depending on the participant’s preference. Semi-structured interviews allow for the exploration of sensitive topics and were considered the most appropriate data gathering tool within this context [17].

### Recruitment and participants

Participants over the age of 18 years who had initiated GAMT over five years ago were invited to take part in the study. Recruitment took place via advertising over social media, through the trans-led charity *Gendered Intelligence* (UK) and snowball sampling. Details of the study were circulated inviting those eligible and interested to contact the researcher directly. Prior to the interview and again during the interview it was verbally confirmed that participants had initiated GAMT over 5 years ago. Participants were recruited over six months between December 2019 and May 2020. The sample size was determined by data saturation whereby recruitment continued until no new information was provided in the interviews [18].

Saturation was reached after interviewing 23 participants (video calls *n* = 17, face-to-face *n* = 6). The mean age of those interviewed was 45.57 years (*SD* = 16.37, range 22–76). All participants were White. Table 1 shows the demographic information of the participants.

**Table 1:** Participant demographics

Demographic	Category				
Employment	Employed	Retired	Education	Disabled and not working	Homemaker
*n* (%)	11 (47.83%)	5 (21.74%)	3 (13.04%)	2 (8.7%)	1 (4.35%)

Level of Education	BA/BSc or equivalent	MSc/MA or equivalent	A level or equivalent	GCSE or equivalent	PhD or equivalent
*n* (%)	7 (30.43%)	6 (26.09%)	5 (21.74%)	3 (13.04%)	2 (8.7%)

Sexuality	Gay/lesbian	Straight	Bisexual/pansexual	Questioning	Other (asexual/panromantic asexual /queer/queer dyke)
*n* (%)	6 (26.09%)	5 (21.74%)	5 (21.74%)	1 (4.35%)	5 (21.74%)

Relationship status	In a relationship(s)	Single	Divorced/separated	Married	Widowed
*n* (%)	9 (39.13%)	4 (17.39%)	4 (17.39%)	5 (21.74%)	1 (4.35%)

Religion/spiritual belief	No religion	Christian	Pagan	Buddhist	Other (Wiccan/Agnostic/Humanist)
*n* (%)	11 (47.83%)	3 (13.04%)	3 (13.04%)	2 (8.7%)	4 (17.39%)

Gender identity	Trans woman/woman/woman with trans history	Trans man/man	Gender diverse (nonconforming/agender)		
*n* (%)	15 (65.22%)	6 (26.09%)	2 (8.7%)		

Ethnicity	White British	White other			
*n* (%)	17(73.91%)	6(26.09%)			

## Materials

### Demographic questionnaire

Before the interview, participants completed a demographics questionnaire that included general items asking the participant’s age, gender identity, sex assigned at birth, employment status, education level, sexual orientation, relationship status, and religion.

### Interview guide

The interview guide was developed by drawing upon the relevant academic literature as well as discussion with the researchers and members of the Service Users Research Advisory Group (patients or previous patients who are consulted on research activities) [19]. It contained open-ended questions allowing participants to identify and describe factors which impacted their wellbeing, such as “What are the factors that have an impact on your life satisfaction currently?” and follow up prompts such as “What makes you feel happy with your life currently?” or “Is there anything that makes you feel unhappy with your life currently?” (Table 2). The interviews were conducted by the same researcher and were recorded with the knowledge and consent of the participants. This researcher identifies as nonbinary and so was an ‘insider researcher’ [20]. In this context, insider researcher refers to the researcher being a member of the population under study meaning that they shared an experiential understanding with the participants [21].

**Table 2:** Interview guide: prompts are written in italics and are used only if the participant is having difficulty providing answers

Section of interview	Question
Section 1	How long ago did you start gender treatment? Are you currently being seen by gender services? If not, why is this the case? *Prompt: If discharged, when and why did this happen?*
Section 2	How satisfied are you with your life at the moment? What are the factors that have an influence on your life satisfaction currently? *Prompt: What makes you feel happy with your life?* *Prompt: Within your relationships (partners/family/friends)/ work/leisure/personally* *Prompt: Is there anything that makes you feel unhappy with your life?* *Prompt: Within your relationships (partners/family/friends)/work/leisure/personally*
Section 3	Have there been any changes in your life satisfaction since transitioning? *Prompt: What has contributed to these changes?* *Prompt: Have these been—negative changes? Positive changes?*
Section 4	Could you tell me about any challenges you faced during or after your transition? *Prompt: In the past and/or currently?* *Prompt: Can you give me an example of how your transition has had a negative/positive effect on you?* How did you deal with these challenges?
Section 5	Are there any other aspects that you feel are important that have not been discussed? *Prompt: The Service/Personally/Transitioning* */Relationships/Work/Leisure*

## Data analysis

Thematic analysis, using an inductive approach, was employed to analyse the transcripts. Inductive thematic analysis was identified as the most appropriate method for analysing the data as it provides a rich thematic description while allowing the data to lead the analysis in identifying themes, but still facilitates the researcher identifying implicit aspects of the data [18]. The process of thematic analysis was followed [18] in which the data was transcribed and then read several times to ensure familiarity with the data. The first author then analysed the data to identify initial codes and themes using the NVIVO analysis software. These themes and codes were then reviewed by an independent, White, nonbinary researcher and the research team. From this point the themes were defined and named and the report was produced. Themes were considered as potentially prominent when they were consistently discussed across several participants’ interviews.

## Results

Thematic analysis revealed four themes, each with several sub-themes within them (See Table 3).

**Table 3:** Summary of themes and sub-themes

Theme	Sub theme
1: Interactions with healthcare services	1.1 Struggles of accessing appropriate healthcare
	1.2 Benefits of understanding and rapport in healthcare
2: Seeking societal acceptance	2.1 Harmful expectations of gender presentation
	2.2 Feeling unsafe due to a lack of societal acceptance
	2.3 The stress of gender identity disclosure
	2.4 Difficulties in finding a sense of belonging
	2.5 Rewards of promoting trans awareness
3: Quality of social support	3.1 Benefits of having the right kind of social support
	3.2 Harm of support lost due to transition
	3.3 Comfort from LGBTQ + support
4: The ‘double-edged sword’ of media and social media	4.1 The negative impact of transphobia and harassment in media and social media
	4.2 Benefits of connecting through social media and the positive representation in the media

### Theme 1: Interactions with healthcare services

#### Struggles of accessing appropriate healthcare

In this theme participants discussed the issues they experienced with accessing appropriate healthcare both in regards to THS and more generally. Participants who were still in touch with THS acknowledged the current negative impact on their wellbeing of access factors, such as waiting times for gender affirming surgeries, delays or cancellations of surgeries, and poor communication with THS. Several participants who were no longer being treated by THS felt that there was a lack of psychological care for trans people once they had been discharged and wished for more support from THS in this regard.

I'm currently in a little bit of limbo at the moment because I got discharged from [GIC Clinic] without them telling me I had been, and then I've had to go through the referral process again, […] it’s impacted my life satisfaction (Aged 32, Trans Man)

Participants also discussed issues they faced in non-specialist health services. These issues focused on the lack of knowledge of transgender needs held by General Practitioners (GP), leading to patients feeling vulnerable and more at risk of not receiving correct treatment once they were no longer being seen by THS. Due to this lack of knowledge participants also found themselves in the situation of having to explain issues that trans people faced, and even medical care, to these professionals.

After getting discharged it’s a little bit more […] you're feeling a bit more vulnerable to them and their [GPs’] ignorance […]. And I think that is something that is important to discuss for trans wellbeing (Aged 25, Trans Man)

#### Benefits of understanding and rapport in healthcare

Participants identified the benefits to wellbeing of having a GP, THS clinicians, and therapists that respected them and understood issues that transgender people face. The knowledge that these professionals could be relied on to provide appropriate care, with respect, was discussed as a positive factor in participants’ wellbeing. This respect and acknowledgement is also seen in later themes focusing on social support, where again, this had a positive impact on wellbeing.

Being made to feel real calm with a certain GP really respected really centred and […] knowing that I'll always get my hormones is a big thing for me (Aged 30, Non-Conforming)

### Theme 2: Seeking societal acceptance

#### Harmful expectations of gender presentation

Expectations of gender presentation was a recurring theme impacting on wellbeing. One participant discussed the negative impact of the conflict of being expected to not care about passing but also finding that passing was something many still want and struggle with. This issue of passing and the expectations of others and oneself was explored further by a participant who spent some time at a trans-only group and discussed the negative impact of attempting to have their gender identity accepted and validated in a cis-normative society. Participants discussing passing and expectations of gender presentation in a cis-normative society imply an additional factor to navigate to be accepted.

Just be kind of immersed in trans culture […] you don't really realise how much of an effort you're making […] to pass until you don't suddenly don’t have to […] it was sort of eye opening in a sense to be like oh shit I'm actually doing myself a disservice (Aged 22, Trans Man)

Some participants discussed experiencing ‘passing privilege’ (the privileges inherent in being able to appear cisgender). While this had a positive impact on their life satisfaction, they experienced problems in certain spaces where their passing privilege could be revoked due to cis-normative expectations of bodies.

I feel like, you know, very secure in a lot of situations but put me in men-only space and it feels different, and my passing privilege could be revoked in an instant because my body doesn’t fit certain requirements (Aged 31, Trans Man)

Other issues were raised particularly from a gender diverse participant’s experience in which they experienced invalidation from within the trans community due to other trans people’s expectations of gender presentation. This experience highlights issues even in trans spaces where binary body expectations are placed on those who identify outside of the binary.

I still get asked when I'm transitioning, and in trans spaces I still get asked […] ‘When are you gonna get on hormones then’? […] and that's so frustrating and so like invalidating and like that hurts so much (Aged 30, Non-Conforming)

#### Feeling unsafe due to a lack of social acceptance

Safety when socializing was also noted as a factor in wellbeing. Some participants pointed out that they felt they had to actively plan or curtail their socialising based on concerns for their safety. One participant echoed this but from a position where they felt they could physically defend themselves, thus giving them the confidence to engage in social activities without fear.

Back home I used to go to pubs and stuff all the time um I don't do that anymore […] I don't know where we're going, I've not been there before I have no idea if I'm physically safe going there (Aged 35, Trans Woman)

#### The stress of gender identity disclosure

Several participants also covered choosing if, how, and when to come out. Disclosing their trans identity was, for some, a positive experience while for others this was a source of stress, as reactions to the disclosure of identity are rarely certain and it is difficult, if not impossible, to retract once disclosed. In addition, for those who were generally cis-passing, the decision whether to come out or not was a stressful one in of itself as being read as cis provided no validation to their gender identity.

Now that I'm read generally as cis […] if I come out that's a choice that I've made rather than just something that's happened, and it's […] stressful, you know? and then if I've decided not to come out then it's stressful to kind of keep it under wraps and worry about it on your own (Aged 22, Trans Man)

In addition to this, a gender diverse participant discussed the issues around having to come out as agender to be gendered correctly as someone who used they/them pronouns. Thus, in order to have their identity potentially validated they had to put themselves in a vulnerable position.

… It's just a hassle to have to explain it and then […] you basically put the power on that person, and then that person has the power to completely invalidate you or validate you and, yeah, it's good when people validate you but… (Aged 33, Agender)

#### Difficulties in finding a sense of belonging in society

Participants highlighted issues with finding a sense of belonging and the impact this could have. One participant found that being reminded of their transition was something they found uncomfortable and so they avoided these scenarios, stating that they felt no need to be defined by their trans identity exclusively. Another participant discussed that they are still finding their place within the trans community, which was a challenge to their wellbeing as they had been an active member of LGBT women’s groups before transition and were re-evaluating their dynamic with LGBT groups. This led to shifting of both social and support groups in their lives.

I still don't know where I fit in like the larger transworld still, like, I still don't really know where I fit […] So that's probably like my biggest challenge, that's probably the most relevant to, like, life satisfaction (Aged 31, Trans Man)

#### Rewards of promoting trans awareness

Participants identified that promoting awareness of trans issues was important and could be a rewarding experience. This experience was described in various contexts including giving talks on trans experience, engaging in activism, providing information through social media, and discussing issues relevant to trans people with friends, family and significant others. This was discussed not only in regards to informing cis people, but also promoting awareness of these issues and informational resources to other trans people. However, it was noted that raising awareness often felt like a responsibility and could be stressful even when they felt it was worth the cost. In particular, the issue of having to out oneself to discuss this with cis audiences was often referred to as a sacrifice.

It takes me out of my comfort zone to do the awareness stuff because I don't want to rock up to places saying that I'm trans, but it's well worth the sacrifice to give to others to raise that awareness (Aged 44, Man)

### Theme 3: Quality of social support

#### Benefits of having the right kind of social support

One of the most important factors that participants discussed was the importance of having the right social support for them. These relationships were diverse in nature, including familial support, partners, and friends. Often when this social support was discussed it was in terms of providing understanding and validation. This support was not always seen immediately, Participants covered the need to either remove those who were unsupportive or guide them towards understanding, the latter involving a great deal of emotional labour.

I’ve had different periods, my family was, for about six years, not supportive, so with time and prodding and with more time they kind of came around as they do its very sort of so now they provide me with social support (Aged 28, Woman with Trans History)

In addition to familial support, support in more official settings such as the workplace were discussed. A supportive workplace environment was valued, as was having visible policies and anti-discrimination legislation in place to provide more formal protections.

People were incredibly supportive, but I was also conscious that I had the legislation behind me and policy at work so no one could actually give me any crap (Aged 44, Trans Man)

#### Harm of support lost due to transition

Some participants described their experiences of loss of support during their transition due to their trans status. The losses participants described included relationships and connections with friends, family, and significant others. This isolation was often identified as a negative factor in their wellbeing and reflects the impact of a lack of validating social support.

So I've lost […] through my transition most of my friends, family. Only my mum speaks to me now and well I'm not exactly convinced that she's happy with it, but she's accepted at least (Aged 50, Trans Woman)

An additional issue which was discussed in relation to the loss of significant others was the loss of a safe home. Some participants were forced to leave their home or remain living in a hostile environment. While this often started at the point of coming out, this continued to impact on participants’ current wellbeing due to the significance and often their long-lasting consequences, including rebuilding social networks.

She [ex-partner] kicked me out of the house, so I've gone from being living in a pretty comfortable semi-detached 3-bedroom house to, you know, to almost being, you know, sofa surfing (Aged 60, Woman with Trans History)

#### *Comfort from LGBTQ* + *peer support*

Several participants discussed their connections with other LGBTQ + peers in relation to their wellbeing. One participant highlighted issues with a lack of connection with their peers and how this lack of connection lead to a feeling of isolation. Conversely, many participants highlighted the positive impact of support from the LGBTQ + community and elaborated that the shared experiences that they had in common allowed them to feel comfortable and provided an opportunity for validation. In particular, not having to police their actions or behaviour in regards to gender presentation, as they may do with cis people, was welcomed. The value of peer support is also reflected in the next theme, in which the use of social media to connect with peers is discussed as one of the benefits of using social media.

I’m the only trans person in my department [at work], and also one of the few queer people in my department and […] I’m definitely sort of on the minority and so I’m sort of on my own little bizarre little island (Aged 31, Trans Man)

The LGBT identity is really important as well because it means that I can totally be myself I don't need to feel worried or, I don't know, police my actions or behaviour in terms of not being a masculine enough or whatever (Aged 32, Trans Man)

You don't have to go through that process with these people [trans people] they know the score they've gone through it, and it just makes things a lot easier at times (Aged 29, Trans Woman)

### Theme 4: The ‘double-edged sword’ of media and social media

#### The negative impact of transphobia and harassment in media and social media

The experiences of transphobia and harassment in media and social media were identified as factors in participants’ wellbeing. Negative representation and discussion around trans people and trans rights in the media, which reflected the views of some cis people, had a detrimental impact upon wellbeing. These negative representations caused some participants to distance themselves from certain media sources and ensure that they were in a positive mental health state before engaging with media. This need to distance themselves from or assess their current wellbeing prior to engaging with media is an additional layer of emotional labour impacting on wellbeing. Several participants discussed personal experiences of harassment on social media which forced to them to leave those platforms or open up new accounts. One participant highlighted that they were now much more careful about opening up online. This understandable caution would likely make it more difficult to develop online social support networks.

I'm very selective about the sort of media that I can see you and there's barely any mainstream media now that stuff is yeah, I leave it to the professionals really (Aged 43, Woman)

Started getting, like, negative messages and private messages on Twitter […] to the point that I just needed to start those over again, […] I've started being a lot more careful about opening up and so I wouldn't get attacked (Aged 41, Woman)

#### Benefits of connecting through social media and the positive representation in the media

There was, however, some discussion of the positive impact of social media and media. Social media provided the opportunity to network and socialize with other trans people. This facilitated peer support and allowed for swapping of information about resources available. Participants not only discussed the positives of social media connections but also positive trans representation in the media. LGBTQ+, and in particular trans, characters being represented in ways that did not focus on trauma or tragedy were seen as much needed validation in media.

I suppose social media being a double-edged sword on the one hand it has got this horrible negative elements but on the other hand twitter and Instagram stuff have given me the chance to meet other trans people so I probably wouldn't have had in real life (Aged 32, Trans Man)

Watching stuff even like with small representation in it is somewhat helpful, when it's not all about that person and their tragedy (Aged 33, Agender)

## Discussion

The aim of this study was to identify factors that affect the wellbeing of a population of trans people for whom little is known; those who initiated GAMT sometime ago, what we called ‘later in transition’. Previous studies have mainly focussed on trans people who are in the process of, or who have recently, transitioned [16]. Therefore, this study wanted to explore which factors are, or have remained, important for trans people later on. As such, this study addresses an important gap in the literature; understanding the experiences and needs of people who may no longer be in contact with THS. The interviews conducted for this study have provided novel insight into these factors in this population, while centring trans voices and experiences. The findings suggest that even at this later stage, trans people are negatively impacted by a lack of social acceptance, benefit from strong social support (in particular from those who have shared experience), experience pervasive transphobia in media and social media, and face issues when interacting with THS and non-specialist medical professionals.

In terms of social support, the shared experiences of those attending support groups or receiving support from peers allowed for participants to feel more at ease and able to discuss their issues without the additional emotional labour of explaining nuances of the experience. This supports findings of prior literature in the area which highlights the value and protective nature of peer support for the wellbeing of trans people [22–25]. However, some participants, particularly those who were gender diverse, experienced issues even in trans spaces. These issues were centred on others’ expectations of trans bodies and gender presentation and supports a previous study which found similar exclusionary attitudes towards gender diverse people within trans spaces [26]. These themes highlight the importance of the provision of LGBTQ + support groups, and particularly trans support groups, to facilitate community social support, but inclusiveness and understanding in these spaces particularly for gender diverse people is vital. This is particularly important to the trans community in view of the possibility of a loss of social support when transitioning highlighted in this and other studies [22–24, 26].

Social media is an important tool for communication and contact with others [22, 23], however this was discussed as a ‘double-edged sword’ for some participants. Social media, and the media in general, were identified as having a significant detrimental impact. Online harassment through social media, due to being open about one’s trans status and experiences, was not uncommon and had an understandable negative impact on wellbeing. In addition, negative coverage of trans issues by the media was not only detrimental to participants’ wellbeing but also changed media consumption habits, leading to caution in the media sources consumed to avoid transphobic content. The additional stress of avoiding those media outputs also impacted on wellbeing via the distress associated with learning that a previously thought safe media source is no longer safe. Possible future research could focus on examining how the wellbeing of trans people interacts with other key factors, such as age, in the utilization of social media and media by trans people.

In addition to deeper exploration of previously examined topics, the study has identified other under-explored topics not covered in the current literature. In particular, the vulnerability felt by trans people due to a lack of knowledge held by non-specialist medical professionals [25, 27]. This was exacerbated by poor communication and early discharge from THS. This leaves trans people in the vulnerable position of seeking care for GAMT-related health needs, ranging from post-surgical care to hormone level monitoring, from non-specialist medical professionals who, in many cases, lack the resources and understanding to provide comprehensive care. Acquiring GAMT and related care can be a personal and stressful experience and a lack of understanding and clear, regular communication from THS and non-specialist medical professionals can only make the experience more difficult and impact negatively on wellbeing.

To best centre trans voices and experiences in this study, trans and gender diverse people were included throughout the process as part of the research team, including the lead researcher. This is clearly a benefit and one participant mentioned that in discussing issues with other trans people “*they know the score they've gone through it and it just makes things a lot easier at times”*. This study provides data that describes the real lived experiences of a population that has not been previously well represented in research. However, it is recognised that despite attempts during recruitment to diversify demographics in participants the sample lacked diversity in terms of ethnicity and a more diverse sample is likely to bring forward new themes or nuances to the existing themes.

Overall, the narrative of support and acceptance is a prominent one in the outcomes of this study, with participants using their own lived experience to discuss how support, acceptance, and/or its absence has impacted on their wellbeing. Using this new information from participants’ experiences we can now provide recommendations for specialist and non-specialist healthcare providers and showcase not only the importance of third-party support organisations that provide trans spaces, but also protective legislations, more ethical reporting of trans issues in the media, and awareness in the general public. Each of these aspects emphasise the value for trans people of formal and informal support as well as societal acceptance and the positive impact that these can have on life satisfaction and wellbeing.
